# Factors Associated With Delayed and Late Initiation of Antiretroviral Therapy Among Patients With HIV in Beijing, China, 2010–2020

**DOI:** 10.3389/ijph.2023.1605824

**Published:** 2023-06-21

**Authors:** Yuanqi Mi, Mengge Zhou, Yuhong Zeng, Peicheng Wang, Liangmin Gao, Feng Cheng

**Affiliations:** ^1^ Department of Epidemiology, Bloomberg School of Public Health, Johns Hopkins University, Baltimore, MD, United States; ^2^ Department of Epidemiology and Biostatistics, Institute of Basic Medical Sciences, Chinese Academy of Medical Sciences, Beijing, China; ^3^ Department of Epidemiology and Biostatistics, School of Basic Medicine, Peking Union Medical College, Beijing, China; ^4^ Department of Epidemiology, College of Preventive Medicine, Army Medical University (Third Military Medical University), Chongqing, China; ^5^ Vanke School of Public Health, Tsinghua University, Beijing, China; ^6^ Institute for International and Areas Studies, Tsinghua University, Beijing, China

**Keywords:** associated factors, China, antiretroviral therapy, HIV, CD4

## Abstract

**Objectives:** To determine factors associated with late and delayed antiretroviral therapy (ART) initiation in China and provide evidence for HIV prevention.

**Methods:** Logistics regression model was used to determine factors associated with three outcomes: late (CD4 cell count <200 cells/µL or clinical AIDS diagnosis prior to ART initiation), delayed (more than 1 month between HIV diagnosis date and ART initiation) and either late or delayed ART initiation.

**Results:** Multivariable analysis revealed that male, heterosexual, HIV diagnosis before 2014, HBV/HCV seropositive, and tuberculosis were associated with increased odds of all three outcomes. Conversely, married or cohabiting patients were less likely to have delayed ART initiation and either late or delayed ART initiation, while people who inject drugs were more likely to have these two outcomes. Additionally, older age was associated with an increased risk of having either late or delayed ART initiation, but a decreased risk of delayed ART initiation.

**Conclusion:** The proportion of late and delayed ART initiation decreased significantly after the release of the 2016 guidelines in China. To further improve late diagnosis and early treatment, precise interventions for key populations are required.

## Introduction

At the end of 2020, the number of people living with HIV(PLWH) has reached 37.7 million globally and 1.5 million new HIV cases were diagnosed that year [1]. In China, there were 1.053 million PLWH in 2020. Early ART initiation could reduce AIDS-related events and virological failure [2, 3]. WHO first proposed the “Treatment as Prevention” strategy in 2013 and recommended the immediate provision of ART to all PLWH regardless of their CD4 count [4]. China has made policy responses accordingly, changing the CD4 count threshold of ART initiation to no threshold in 2016 [5, 6]. However, patients’ inactive diagnosis and not initiating ART immediately remain two major issues [1] which caused serious difficulties in HIV/AIDS prevention and control.

Late ART initiation and delayed ART initiation are two outcomes used in previous clinical studies. International studies have mostly used AIDS-related clinical events (late presenters, LPs) [7] or CD4 cell count level (<200 cells/μL [7–9], <350 cells/μL [10], <500 cells/μL [11]) at ART initiation as an operational definition of late ART initiation, and used the lag time (1 month [5], 3 months [12, 13] and 12 months [11]) between HIV diagnosis and ART initiation to define delayed ART initiation. Previous studies have rarely compared different outcomes. In China, gender, transmission routes, age, and year of ART initiation were associated with late ART initiation [14–16], and marital status was found to be associated with delayed ART initiation [5]. Before 2016, the situation of ART initiation in China has been improving despite the challenges of inactive ART initiation remained [5]. Therefore, a detailed study of the latest situation of late and delayed ART initiation since China’s recent treatment guidelines reformation (2016) is a necessity to better implement national AIDS prevention and control programs.

In this study, we aim to explore associated factors of late and delayed initiation of ART and describe the clinical data of patients who initiated ART in Beijing, China by analyzing the trends in the median CD4 cell counts and median lag time from the past 10 years. This study adds a unique perspective to the literature by including different outcomes to define treatment positivity and concludes a more detailed and realistic description of clinical data.

## Methods

### Data Collection and Study Design

The Beijing Center for Diseases Prevention and Control routinely collected clinical data of HIV-infected individuals through a standard web-based data collection platform. Three hospitals in Beijing, Beijing You’an Hospital, Beijing Ditan Hospital and 302 Military Hospital of China, designated for HIV treatment, would report data by clinicians. All data were a direct reporting of hospital electronic medical record information on the date of ART prescription, which was of good accuracy and quality. The required data included baseline characteristics, laboratory measurements, co-infections, opportunistic infections and ART regimen, et cetera. Between 1st January 2010 and 31st December 2020, a total of 23,919 HIV-infected individuals initiating ART in Beijing, China, were included in this study. Patients with missing values of gender, age, marital status, infection types, time of HIV diagnosis, time of ART initiation, clinical manifestation of HIV, opportunistic infection, HBV/HCV seropositive and Tuberculosis were excluded (*n* = 3,227). Finally, a total of 20,692 patients were included in this study. Confidentiality was protected as part of the management of individual information and the processing of personal data. The study protocol was approved by the Research Ethics Committee of the Beijing Center for Diseases Prevention and Control. No informed consent was required.

### Definition of Variables

Marital status included single, married or lives with partner, divorced or separated and widowed. Infection type included homosexual, heterosexual, people who inject drugs (PWID) and others. Body mass index (BMI) was calculated as weight in kilograms divided by height squared in meters. The laboratory testing included CD4 count and HIV viral load at ART initiation. Lag time was defined as the period between HIV diagnosis and ART initiation. Year of diagnosis when divided into three groups based on guidelines influence. Since the study population was diagnosed after 2010, we only included a total of two guidelines from this period, which were published on 13 May 2014 and 15 June 2016. Clinical manifestation of HIV was defined as a patient with one of the following symptoms at the initiation of ART: fever, cough, expectoration, dyspnea, chest pain, night sweats, diarrhea, nausea, projectile vomiting, headache, decreased vision, blurred vision, rash, swollen lymph nodes. Opportunistic infection was defined as a patient with at least one opportunistic infection within 3 months before ART initiation. HBV/HCV seropositive was defined as a patient with HBV or HCV coinfection at the initiation of ART. Tuberculosis was defined as a patient with tuberculosis within 1 year before ART initiation. Co-infection with hepatitis B virus (HBV) or hepatitis C virus (HCV), tuberculosis, clinical manifestation of HIV, opportunistic infections, and WHO HIV clinical staging were judged by clinicians.

### Definition of Late ART Initiation and Delayed ART Initiation

There are three outcomes used in this study. The first one is “late ART initiation,” which was defined as having a CD4 cell count <200 cells/µL or having a clinical AIDS diagnosis at ART initiation (including having current clinical syndromes, opportunistic infections 3 months before ART initiation or at WHO stage III or IV), a definition used in four studies in China [14–17]. The second one is “delayed ART initiation,” which provided an alternate measure of timely ART initiation based on self-reported HIV diagnosis date, and is defined as having a period of more than 1 month between HIV diagnosis date and ART initiation, a definition used in two national studies in China [5, 18]. The last one is “either late or delayed ART initiation” and was defined as having at least one of the above outcomes.

### Statistical Analysis

Continuous variables with normal distribution were reported as mean (standard deviation), and differences between groups were compared using t-tests; continuous variables with skewed distribution were reported as median (interquartile range [IQR]); and all the categorical variables were presented as the number (percentage) and compared using chi-square test. We conducted a logistic regression model to analyze the associated factors of late ART initiation, delayed ART initiation and either late or delayed ART initiation. The predictors included in the multivariable logistic regression model were selected based on a significance level of *p* < 0.20 in the univariable logistic regression model and reference to past research designs. We included the following candidate variables: gender (binary variables: male/female), age (categorical variables: ≤24, 25–34, 35–45, >45), BMI (categorical variables: ≤18.4, 18.5–24.9, 25–29.9, >30), marital status (categorical variables: single, married/cohabitating, divorced/separated, widowed), infection type (categorical variables: homosexual, heterosexual, PWID, other), year of diagnosis (categorical variables: before 2014, 2014 to 2016, after 2016), lag time group (categorical variables: ≤1 month, 1–6 months, 6–12 months, >12 months), CD4 count at ART initiation (categorical variables: ≤200 cells/μL, 201–350 cells/μL, 351–500 cells/μL, >500 cells/μL), opportunistic infection (binary variables: with/without), clinical manifestation of HIV (binary variables: with/without), HBV/HCV seropositive (binary variables: with/without) and tuberculosis (binary variables: with/without). Statistical analyses were performed using SPSS 23.0 (IBM SPSS, Inc., Armonk, NY, United States). Two-tailed *p* values of less than 0.05 were considered statistically significant.

## Results

### Baseline Characteristics of Patients at ART Initiation

A total of 20,692 HIV-infected patients with 6,733 (32.5%) defined as late ART initiation (late group), 9,641 (46.6%) defined as delayed ART initiation (delayed group) and 13,534 (65.4%) defined as either late or delayed ART initiation receiving initial ART between 2010 and 2020 were included. The mean (SD) age of the 20,692 participants was 33.2 (10.2) years, and most patients were male and single. More than half of the patients (56.1%) had a BMI within the normal range (18.5–24.9). The most common transmission route was homosexual (88.4%), followed by heterosexual (10.5%) and people who inject drugs (PWID) (0.6%). 43.9% of the patients who initiated ART were diagnosed with HIV after 2016, and 27.2% before 2014. The mean (SD) of plasma HIV RNA load is 4.4 (0.9) log10 copies/mL and the mean (SD) of CD4 count at ART initiation is 312.5 (194.6). 34.6% of patients initiated ART with a CD4 at 201–350 cells/μL. The most common WHO HIV clinical staging among patients is stage I (79.3%). Most patients initiated ART 1–6 months from their HIV diagnosis date (53.4%). Only 4.5% initiated ART with clinical manifestation of HIV, 4.2% with at least one opportunistic infection within 3 months before ART initiation, 5.7% with HBV/HCV seropositive, and 0.7% with tuberculosis. In addition to the plasma HIV RNA load, the rest of the variables were all significantly different between the late group and the non-late group (*p* < 0.001). Apart from the clinical manifestation of HIV and opportunistic infection, the rest of the variables were all significantly different in the delayed and non-delayed group (*p* < 0.001). The common factors of both outcomes are gender, age, BMI, marital status, transmission routes, year of diagnosis, HBV/HCV seropositive status and tuberculosis (Table 1).

**TABLE 1:** Characteristics of patients at ART initiation, (Beijing, 2010-2020).

	Total (*n* = 20,692)	Non-late ART initiation (*n* = 13,959)	Late ART initiation^i^ (*n* = 6,733)	*p*-value	Non-delayed ART initiation (*n* = 11,051)	Delayed ART initiation^j^ (*n* = 9,641)	*p*-value
Female, n (%)	772 (3.7)	460 (3.3)	312 (4.6)	<0.001	474 (4.3%)	298 (3.1%)	<0.001
Age, year, mean (SD)	33.2 (10.2)	32.1 (9.6)	35.6 (10.9)	<0.001	34.0 (10.6)	32.4 (9.6)	<0.001
Age groups, year, n (%)				<0.001			<0.001
≤24	4,607 (22.3)	3,541 (25.4)	1,066 (15.8)		2,318 (21.0)	2,289 (23.7)	
>25 to ≤34	9,633 (46.6)	6,654 (47.7)	2,979 (44.2)		4,953 (44.8)	4,680 (48.5)	
>35 to ≤45	3,728 (18.0)	2,286 (16.4)	1,442 (21.4)		2,071 (18.7)	1,657 (17.2)	
>45	2,724 (13.2)	1,478 (10.6)	1,246 (18.5)		1,709 (15.5)	1,015 (10.5)	
BMI^a^, kg/m^2^, n (%)				<0.001			<0.001
≤18.4	1,586 (7.7)	839 (6.0)	747 (11.1)		915 (10.3)	671 (9.3)	
>18.5 to ≤24.9	11,614 (56.1)	7,873 (56.4)	3,741 (55.6)		6,320 (71.4)	5,294 (73.4)	
>25 to ≤29.9	2,455 (11.9)	1,811 (13.0)	644 (9.6)		1,382 (15.6)	1,073 (14.9)	
>30	409 (2.0)	313 (2.2)	96 (1.4)		235 (2.7)	174 (2.4)	
Marital status, n (%)				<0.001			<0.001
Single	14,761 (71.3)	10,404 (74.5)	4,357 (64.7)		7,562 (68.4)	7,199 (74.7)	
Married/cohabitating	4,750 (23.0)	2,869 (20.6)	1,881 (27.9)		2,771 (25.1)	1,979 (20.5)	
Divorced/separated	1,085 (5.2)	633 (4.5)	452 (6.7)		655 (5.9)	430 (4.5)	
Widowed	96 (0.5)	53 (0.4)	43 (0.6)		63 (0.6)	33 (0.3)	
Transmission route, n (%)				<0.001			<0.001
Homosexual	18,283 (88.4)	12,569 (90.0)	5,714 (84.9)		9,718 (87.9)	8,565 (88.8)	
Heterosexual	2,163 (10.5)	1,261 (9.0)	902 (13.4)		1,261 (11.4)	902 (9.4)	
PWID	129 (0.6)	83 (0.6)	46 (0.7)		18 (0.2)	111 (1.2)	
Other	117 (0.6)	46 (0.3)	71 (1.1)		54 (0.5)	63 (0.7)	
Year of diagnosis^b^, n (%)				<0.001			<0.001
Before 2014	5,626 (27.2)	3,389 (24.3)	2,237 (33.2)		1,368 (12.4)	4,258 (44.2)	
2014 to 2016	5,992 (29.0)	4,200 (30.1)	1,792 (26.6)		2,747 (24.9)	3,245 (33.7)	
After 2016	9,074 (43.9)	6,370 (45.6)	2,704 (40.2)		6,936 (62.8)	2,138 (22.2)	
Plasma HIV RNA load^c^, lg copies/mL, mean (SD)	4.4 (0.9)	4.3 (0.8)	4.8 (0.8)	0.064	4.5 (0.9)	4.4 (0.8)	<0.001
CD4 count at ART initiation,/μL, mean (SD)	312.5 (194.6)	398.7 (161.3)	133.7 (122.6)	<0.001	301.0 (197.9)	325.7 (190.0)	<0.001
CD4 count at ART initiation, counts/μL, n (%)				—			<0.001
≤200	5,708 (27.6)	—	—		3,437 (31.1)	2,271 (23.6)	
>201 to ≤350	7,164 (34.6)	—	—		3,646 (33.0)	3,518 (36.5)	
>351 to ≤50	4,825 (23.3)	—	—		2,423 (21.9)	2,402 (24.9)	
>500	2,995 (14.5)	—	—		1,545 (14.0)	1,450 (15.0)	
WHO HIV clinical staging, n (%)				—			<0.001
I	16,416 (79.3)	—	—		8,955 (81.0)	7,461 (7.4)	
II	2,149 (10.4)	—	—		979 (8.9)	1,170 (12.1)	
III	1,009 (4.9)	—	—		469 (4.2)	540 (5.6)	
IV	1,118 (5.4)	—	—		648 (5.9)	470 (4.9)	
Lag time groups^d^, month, n (%)				<0.001			—
≤1	1,257 (6.1)	951 (6.8)	306 (4.5)		—	—	
>1 to ≤6	11,051 (53.4)	7,158 (51.3)	3,893 (57.8)		—	—	
>6 to ≤12	5,927 (28.6)	4,093 (29.3)	1,834 (27.2)		—	—	
>12	2,457 (11.9)	1,757 (12.6)	700 (10.4)		—	—	
Clinical manifestation of HIV^e^, n (%)	926 (4.5)	—	—	—	512 (4.6)	414 (4.3)	0.24
Opportunistic infection^f^, n (%)	873 (4.2)	—	—	—	488 (4.4)	385 (4.0)	0.131
HBV/HCV seropositive^g^, n (%)	1,174 (5.7)	713 (5.1)	461 (6.8)	<0.001	553 (5.0)	621 (6.4)	<0.001
Tuberculosis^h^, n (%)	155 (0.7)	20 (0.1)	135 (2.0)	<0.001	48 (0.4)	107 (1.1)	<0.001

^a^ 4,628 individuals did not have a BMI measurement.

^b^ Year of diagnosis when divided into three groups based on guidelines influence. Since the study population was diagnosed after 2010, we only included a total of two guidelines from this period, which were published on 13 May 2014 and 15 June 2016.

^c^ 4,275 individuals did not have a Plasma HIV RNA load measurement.

^d^ Lag time was defined as the period between HIV diagnosis and ART initiation.

^e^ Clinical manifestation of HIV was defined as a patient with one of the following symptoms at the initiation of ART: fever, cough, expectoration, dyspnea, chest pain, night sweats, diarrhea, nausea, projectile vomiting, headache, decreased vision, blurred vision, rash, swollen lymph nodes.

^f^ Opportunistic infection was defined as a patient with at least one opportunistic infection within 3 months before ART initiation.

^g^ HBV/HCV seropositive was defined as a patient with HBV or HCV coinfection at the initiation of ART.

^h^ Tuberculosis was defined as a patient with tuberculosis within 1 year before ART initiation.

^i^ Late ART initiation is defined as having a CD4 cell count <200 cells/mm^3^ or having a clinical AIDS diagnosis at ART initiation (including having current clinical syndromes, opportunistic infections 3 months before ART initiation or at WHO stage III or IV).

^j^ Delayed ART initiation is defined as having a period of more than 1 month between HIV diagnosis date and ART initiation.

### CD4 Counts at ART Initiation and Lag Time Between HIV Diagnosis and Initiation of Antiviral Therapy

In 2010, the median (IQR) CD4 cell counts at ART initiation among the overall patients were 145.5 (39.0, 221.0) cells/μL. From that time on, the CD4 cell counts of the overall patients who initiated ART significantly increased (P for trend = 0.001). And the lag time between HIV diagnosis and ART initiation significantly decreased from 35 (19,75) days to 14 (7,33) days during the 10 years (P for trend = 0.001). The proportion of late ART initiation, delayed ART initiation and either late or delayed ART initiation were 52.5%, 85.0%, and 97.7% in 2010 and decreased steadily to 30.1%, 17.7%, and 41.7%, respectively in 2020 (with the P for trend = 0.002, <0.001 and <0.001) (Table 2).

**TABLE 2:** CD4 counts at ART initiation and lag time between HIV diagnosis and initiation of antiviral therapy, (Beijing, 2010-2020).

Year of ART initiation	CD4 count at ART initiation, count/μL, median (IQR)	Late ART initiation^a^, % (n/N)	Lag time, day, median (IQR)	Delayed ART initiation^b^, % (n/N)	Either late or delayed ART initiation, % (n/N)
2010	145.5 (39.0, 221.0)	52.5 (322/613)	35 (19,75)	85.0 (521/613)	97.7 (599/613)
2011	206.0 (119.2, 279.5)	46.4 (464/1,001)	54 (27,152)	82.3 (824/1,001)	94.7 (948/1,001)
2012	225.0 (142.0, 291.0)	39.9 (567/1,420)	60 (27,206)	77.8 (1,105/1,420)	92.5 (1,314/1,420)
2013	284.0 (185.3, 363.0)	34.9 (643/1,845)	53 (25,222)	71.4 (1,317/1845)	85.0 (1,568/1,845)
2014	304.0 (201.3, 412.0)	32.0 (747/2,335)	44 (22,182)	63.4 (1,481/2,335)	78.9 (1842/2,335)
2015	322.0 (206.3, 429.0)	30.8 (844/2,741)	42 (22,153)	58.0 (1,591/2,741)	74.3 (2,037/2,741)
2016	324.0 (208.0, 447.5)	27.3 (714/2,614)	27 (13,83)	36.9 (965/2,614)	56.2 (1,468/2,614)
2017	310.0 (193.0, 444.2)	28.6 (728/2,542)	20 (9,51)	28.0 (711/2,542)	49.5 (1,259/2,542)
2018	311.0 (189.0, 439.0)	30.9 (722/2,333)	15 (8,34)	21.4 (499/2,333)	46.5 (1,085/2,333)
2019	313.5 (185.0, 446.0)	30.3 (582/1,918)	15 (8,33)	20.4 (392/1,918)	44.8 (859/1,918)
2020	306.9 (180.0, 434.0)	30.1 (400/1,330)	14 (7,33)	17.7 (235/1,330)	41.7 (555/1,330)
*P* for trend	0.001	0.002	0.001	<0.001	<0.001

^a^ Late ART initiation is defined as having a CD4 cell count <200 cells/mm^3^ or having a clinical AIDS diagnosis at ART initiation (including having current clinical syndromes, opportunistic infections 3 months before ART initiation or at WHO stage III or IV).

^b^ Delayed ART initiation is defined as having a period of more than 1 month between HIV diagnosis date and ART initiation.

### Factors Associated With Late ART Initiation

Factors associated with late ART initiation were explored by univariable analysis (Supplementary Table S1). All Factors evaluated in the univariable logistic regression model were further included in the multivariable logistic regression model (Table 3). Female patients were less likely to initiate ART late than males (OR 0.82, 95% CI 0.68–0.98). Patients with a BMI below 18.5 (vs. BMI = 18.5 to 24.9 OR 2.18, 95% CI 1.95–2.44), with HBV/HCV seropositive (vs. without; OR 1.24, 95% CI 1.09–1.41) and tuberculosis (vs. without; OR 11.73, 95% CI 7.25–18.96) were more likely to initiate ART late. Compared with patients younger than 24 years old, those who were 25–34, 35–45, and >45 years old were 1.6-, 2.2-, and 3.0-fold more likely to receive late initiation of ART, respectively. Compared with patients diagnosed with HIV before 2014, those who were diagnosed between 2014 and 2016 and after 2016 were 0.5- and 0.4- fold less likely to receive late initiation of ART, respectively. Compared with patients who have a lag time of 6–12 months, those who have a lag time of below 1 month, 1–6 months, and more than 12 months were 2.2-, 1.5- and 1.1- fold more likely to receive late initiation of ART, respectively (Table 3).

**TABLE 3:** Factors associated with late ART initiation, delayed ART initiation and either late or delayed ART initiation, (Beijing, 2010-2020).

Covariate	Late ART initiation^a^		Delayed ART initiation^b^		Either late or delayed ART initiation	
	OR (95% CI)	*p*-value	OR (95% CI)	*p*-value	OR (95% CI)	*p*-value
Gender						
Male	References	—	References	—	References	—
Female	0.82 (0.68, 0.98)	0.031	0.75 (0.62, 0.91)	0.004	0.76 (0.62, 0.93)	0.006
Age groups, year						
≤24	References	—	References	—	References	—
>25 to ≤34	1.58 (1.45, 1.72)	<0.001	0.95 (0.88, 1.03)	0.252	1.25 (1.16, 1.36)	<0.001
>35 to ≤45	2.22 (1.99, 2.48)	<0.001	0.87 (0.78, 0.97)	0.014	1.45 (1.30, 1.62)	<0.001
>45	3.03 (2.67, 3.44)	<0.001	0.82 (0.72, 0.94)	0.004	1.83 (1.60, 2.09)	<0.001
BMI, kg/m^2^						
>18.5 to ≤24.9	References	—	References	—	References	—
≤18.4	2.18 (1.95, 2.44)	<0.001	0.86 (0.76, 0.97)	0.016	1.43 (1.26, 1.61)	<0.001
>25 to ≤29.9	0.66 (0.60, 0.73)	<0.001	1.04 (0.95, 1.16)	0.396	0.83 (0.75, 0.91)	<0.001
>30	0.68 (0.54, 0.86)	0.002	1.19 (0.96, 1.49)	0.119	0.90 (0.73, 1.12)	0.349
Missing	0.90 (0.84, 0.98)	0.011	0.82 (0.76, 0.89)	<0.001	0.85 (0.78, 0.92)	<0.001
Marital status						
Single	References	—	References	—	References	—
Married or cohabitating	0.97 (0.89, 1.06)	0.549	0.77 (0.70, 0.84)	<0.001	0.82 (0.74, 0.90)	<0.001
Divorced or separated	1.14 (0.99, 1.32)	0.061	0.88 (0.76, 1.03)	0.113	1.05 (0.90, 1.23)	0.509
Widowed	0.98 (0.64, 1.50)	0.926	0.71 (0.43, 1.15)	0.160	0.59 (0.37, 0.93)	0.024
Infection type						
Homosexual	References	—	References	—	References	—
Heterosexual	1.39 (1.24, 1.56)	<0.001	1.06 (0.93, 1.19)	0.395	1.24 (1.10, 1.41)	0.001
PWID	1.15 (0.78, 1.69)	0.495	6.53 (3.84, 11.10)	<0.001	4.73 (2.43, 9.22)	<0.001
Other	2.00 (1.34, 2.98)	<0.001	0.81 (0.54, 1.21)	0.307	1.24 (0.72, 2.13)	0.430
Year of diagnosis						
Before 2014	References	—	References	—	References	—
2014 to 2016	0.56 (0.52, 0.61)	<0.001	0.33 (0.30, 0.36)	<0.001	0.29 (0.26, 0.32)	<0.001
After 2016	0.47 (0.43, 0.51)	<0.001	0.082 (0.075, 0.089)	<0.001	0.10 (0.092, 0.11)	<0.001
Lag time groups, month						
>6 to ≤12	References	—	—	—	—	—
≤1	2.23 (1.93, 2.57)	<0.001	—	—	—	—
>1 to ≤6	1.56 (1.35, 1.81)	<0.001	—	—	—	—
>12	1.09 (0.93, 1.28)	0.301	—	—	—	—
CD4 count at ART initiation, /μL						
>500	—	—	References	—	—	—
≤200	—	—	0.51 (0.46, 0.57)	<0.001	—	—
>201 to ≤350	—	—	0.72 (0.65, 0.79)	<0.001	—	—
>351 to ≤500	—	—	0.88 (0.80, 0.98)	0.018	—	—
Opportunistic infection						
No	—	—	References		—	—
Yes	—	—	0.58 (0.50, 0.69)	<0.001	—	—
HBV/HCV seropositive						
No	References	—	References	—	References	—
Yes	1.24 (1.09, 1.41)	0.001	1.14 (0.99, 1.30)	0.062	1.25 (1.08, 1.45)	0.002
Tuberculosis						
No	References	—	References	—	References	—
Yes	11.73 (7.25, 18.96)	<0.001	2.06 (1.40, 3.04)	<0.001	42.80 (5.95, 308.14)	<0.001

^a^ Late ART initiation is defined as having a CD4 cell count <200 cells/mm^3^ or having a clinical AIDS diagnosis at ART initiation (including having current clinical syndromes, opportunistic infections 3 months before ART initiation or at WHO stage III or IV).

^b^ Delayed ART initiation is defined as having a period of more than 1 month between HIV diagnosis date and ART initiation.

### Factors Associated With Delayed ART Initiation

Factors associated with delayed ART initiation were explored by univariable analysis (Supplementary Table S2). Except for clinical manifestation (*p* > 0.200), all factors evaluated in the univariable logistic regression model were further included in the multivariable logistic regression model (Table 3). In the multivariable analysis, patients who are female (OR 0.75, 95% CI 0.62–0.91), with the marital status of married or cohabitating (vs. single: OR 0.77, 95% CI 0.70–0.84), with a BMI below 18.4 (vs. BMI = 18.5–24.9 OR 0.86, 95% CI 0.76–0.97), with low CD4 count at ART initiation or with opportunistic infection were less likely to delay ART initiation, while PWID (vs. homosexual: OR 6.53, 95% CI 3.84–11.10), patients with HBV/HCV seropositive and with tuberculosis were more likely to delay ART initiation. Patients diagnosed from 2010 to 2020 had a decreased risk for delayed ART initiation, compared with patients diagnosed before 2014, patients diagnosed after 2016 were only 0.10- fold less likely to delay ART initiation (Table 3).

### Factors Associated With Either Late or Delayed ART Initiation

Factors associated with either late or delayed ART initiation (the third outcome) were explored by univariable analysis (Supplementary Table S3). All factors evaluated in the univariable logistic regression model were further included in the multivariable logistic regression model (Table 3). Patients of older age (>45 vs. ≤24 years; 1.83, 95% CI 1.60–2.09) were more likely to have the third outcome, opposite to the results of delayed ART initiation. Similar to the results of late ART initiation, patients with BMI ≤18.4 (OR 0.83, 95% CI 0.75–0.91) were less like to have the third outcome compared with patients with a normal BMI. Married or cohabitating patients were less likely to have delayed ART initiation and either late or delayed ART initiation (OR 0.82, 95% CI 0.74–0.90), but remained a null factor for late ART initiation. Widowed patients were less likely to have the third outcome (OR 0.59, 95% CI 0.37–0.93) compared to single patients, but it was a null factor in the previous analysis. Patients who were male, heterosexual, with earlier years of HIV diagnosis, with HBV/HCV seropositive and with tuberculosis were more likely to have the third outcome, same as the previous analysis. PWID (OR 4.73, 95% CI 2.43–9.22) were more likely to have the third outcome, only consistent with the analysis of delayed ART initiation (Table 3).

## Discussion

To our knowledge, this study is the first analysis of factors associated with both the outcome of late and delayed ART initiation of HIV-positive patients in Beijing. We found that the overall situation of treatment has improved with major challenges still persist.

### Late Diagnosis Remains a Serious Problem That Needs to Be Addressed

In this study, we observed that the median CD4 cell counts at the initiation of ART increased steadily from 2010 to 2020. At the same time, the median lag time of patients and the proportion of patients postponing their treatments decreased significantly, which reflects improvements of the overall ART initiation. This is in line with previous national studies that have been done in Yunnan [5], Shanghai [14], and Taiwan [16], and international studies in Canada [11], Ethiopia [9], Pakistan [12] and Spain [10]. Policy reformation in China contributes to this greatly. In 2003, “Four Frees and One Care” enables the availability of free ART to PWLH [19]. The recommended threshold for CD4 cell counts at ART initiation in the guidelines is also increasing during the past few years [5]. A study in Spain also revealed a trend towards an earlier start of ART was observed during 2015 and 2016 influenced by the last national treatment guidelines recommendations [10].

However, these trends may not solely reflect patient attitudes toward their diagnoses and treatment, but could also reflect the impact from the government’s expanded availability to healthcare. The Chinese CDC has been cooperating with social app since 2013 with the operation of adding HIV testing sites in Beijing, which served approximately 700 MSM per month in 2017 [20]. In 2015, online campaigns were launched, with the testing volume increasing sharply, representing 10 times (2016) and 12 times (2017) the average number of annual tests received during 2013–2014 in Beijing [21]. Guangxi AIDS Conquering Project (GACP) in 2010 also greatly expanded access to HIV testing and ART [22]. Additionally, HIV stigma may lead to avoidance of healthcare services, mental health issues or fear of inadvertent disclosure [23]. The negative behaviors, attitudes of health providers act as enacted stigma towards patients and influences patients’ initiative to healthcare around the world [24, 25]. China was shown to be experiencing a high level of HIV-related stigma in 2014 [26], and expanded culturally appropriate interventions like community-based diffusion of HIV prevention information were proved to be effective in reducing stigma [27], thus improving the overall treatment and testing. Future studies should focus on identifying the barriers to healthcare access that contribute to delayed ART initiation such as transportation, financial barriers, and stigma. The role of health system factors such as healthcare provider attitudes, treatment guidelines, and resource allocation also cannot be neglected in related analyses. In-depth interviews or focus group discussions with patients and healthcare providers are also required to further understand this issue.

In addition, we found that homosexual patients would be more positively engaging in treatments than other patients, compared with no statistical difference in the international studies [10, 11, 13]. The great proportion of homosexual patients in Beijing (88.4%) can account for this difference. Additionally, various interventions towards MSM in China such as pilot work of post-exposure prophylaxis [28, 29] and web-based health education interventions [30] on the related social platforms have resulted in their high awareness of HIV prevention.

We also found that the proportion of patients with late ART initiation decreased mildly from 52.5% in 2010 to 30.1% in 2020, while the proportion of patients with delayed ART initiation decreased drastically from 85.0% in 2010 to 17.7% in 2020. Since delayed ART initiation is measured by the period from HIV diagnosis to treatment initiation, it represents patients’ positivity for treatment and does not contain any information on patients’ diagnoses. Late ART initiation is measured by patients’ clinical indicators at ART initiation, which contain information both on their diagnosis and treatment. This shows that the influence of the guideline has improved the positivity of patients for treatment more than their positivity of diagnosis, consistent with the research conclusion in Yunnan [5]. The situation is similar around the world as the majority of countries released their guidelines after the recommendation by WHO [13]. The study in Northwest Spain also revealed the late HIV diagnosis but earlier ART initiation in the country after its guideline release in 2014 and 2015 recommending treating all HIV-infected patients regardless of their immunological status [10].

We also found that patients with a lag time of less than 1 month were more likely to have late ART initiation, and that patients with low CD4 cell count and opportunistic infection at ART initiation were associated with reduced risk of delayed ART initiation. It shows that most of the patients with strong treatment positivity have late diagnosis, therefore have late ART initiation. The global situation of late diagnosis is severe, with only 84% of people living with HIV knowing their HIV status according to UNAIDS [1]. A study in Spain found that 68.3% had CD4 counts<350 cells/μL at first contact with HIV specialist medical team [10]. Xu et al found that the mean CD4 counts at HIV diagnosis among patients in Yunnan was 324 ± 238 cells/μL in 2016, and concluded that late diagnosis in China remains problematic [5]. Therefore, future research should focus on addressing both behavioral-altering intervention and structural factors study to improve this question.

### Future Policy Direction Based on Analysis From Different Outcomes

Firstly, we found that older patients were more likely to have late ART initiation, which is consistent with the results of Shanghai [14], Ethiopia and Canada [9, 11]. However, the risk of delayed ART initiation has a significant decreasing trend after the age of 35. Younger people are more likely to be diagnosed early, but with a significantly longer lag time. This means older people have the problem of late diagnosis. Awareness-raising interventions can be effective but not enough to address the complex factors that contribute to this phenomenon [31]. Community-based research can help understand the specific challenges faced by this population [32]. Additionally, design and evaluation of interventions are required to consider unique challenges that older patients face, such as co-morbidities, age-related health issues, and social isolation [33]. However, this is contrary to the results in Yunnan [5], and may be related to the composition of HIV transmission patterns in different regions. Patients in Yunnan are mostly PWID, and MSM in Beijing. Future research should expand the diversity of the research location.

We found that patients who are married or cohabitating are less likely to have delayed ART initiation, consistent with the study by Xu et al [5] and Anlay et al [9]. This finding might be associated with the big proportion of patients who were not single among older patients. Heterosexual patients were more likely to have late ART initiation and either late or delayed ART initiation, consistent with the findings in Yunnan, Shanghai and 13 Asian countries [5, 14, 15]. But it was a null factor for delayed ART initiation, similar to the study conducted by Kesselring et al [11]. This means that heterosexual males have higher late diagnosis rate, as proved by the findings in Yunnan [5]. Previous studies [15, 34] have also shown that PWID were more likely to initiate ART late, however, our study found it to be a null factor of late ART initiation. But we found that PWID was more likely to have delayed ART initiation, same as the findings by Sun et al [14]. The small number of PWID (129 patients) in our analysis can result in this difference.

The combined outcome mainly provides a comparison to determine which definition is appropriate for clinical guidance. Being widowed is not significantly associated with the two outcomes independently but with the combined outcome. Consequently, providing solely one or two outcomes is not enough for identifying key populations for further intervention. The negligence of either outcome may cause the overlooking of risk factors.

Lastly, as the lack of patients’ socio-economic characteristics like education, income, and the level of stigma experienced in the database persists, there might be a potential issue of residual confounding on some socially patterned factors like tuberculosis [35]. However, the participants of this study were all HIV patients in Beijing which had relatively high homogeneity. Considering the small proportion of patients with tuberculosis (0.7%), the residual confounding of socio-economic characteristics, although present, was relatively small. Future studies should analyze the impact of socioeconomic characteristics.

### Limitation

There are still some limitations to be considered. First, cross-sectional studies may have the problem of causality inversion. For example, we found that patients with HBV/HCV were more likely to have late ART initiation. With the lack of specific time of HIV infection in the database, we can infer that HBV/HCV seropositive patients could be more likely to initiate ART late or patients’ delay in treatment resulted in a decrease in immunity which led to the infection of HBV/HCV. We could only present all the possible reasons for this phenomenon for future indications. Additionally, the great proportion of males and homosexuals could lead to bias of the results. Plus, some variables have a missing value of more than 10%, so we have to create a separated “missing” group in the regression model. Finally, this database did not include clinical data of patients at diagnosis socio-economic characteristics, we cannot fully describe the status of the late-diagnosed patients and analyze the impact of related factors. However, by using different outcomes, we believe the results can reveal certain problems regarding delayed diagnosis and treatment. Future studies could pay more attention to more detailed characteristics of patients when diagnosed and behavior-related risk factors of late diagnosis to make recommendations with more specificity and feasibility to the general audience.

### Conclusion

This study separately and comprehensively identified factors associated with late and delayed ART initiation, providing indication for HIV prevention in China. The proportion of late and delayed ART initiation was shown to be decreasing significantly after the release of the 2016 guidelines in China, which indicates the impact of well-implemented policies. However, the influence of the guideline has improved the positivity of patients for treatment more than their positivity of diagnosis. The next phase should focus on addressing the structural factors and implementing precise interventions for key populations such as older age groups, heterosexual populations to further improve late diagnosis, and younger age groups, PWID to promote early treatment.
