# Supplementary material for: Factors Associated With Delayed and Late Initiation of Antiretroviral Therapy Among Patients With HIV in Beijing, China, 2010–2020
Source: Int J Public Health. 2023 Jun 21;68:1605824. doi: 10.3389/ijph.2023.1605824 (PMC10321558; doi:10.3389/ijph.2023.1605824)
Supplement: Supplementary file 1 [file Table1.DOCX]

Table 1. Univariate and multiple regression analysis of late ART initiation. Beijing, 2010-2020

| Late ART initiation defined by both CD4 (<200) and AIDS-defining events | | | | |
| --- | --- | --- | --- | --- |
|  | univariate logistic regression | | multiple logistic regression | |
| Covariate | OR (95%CI) | *P* value | OR (95%CI) | *P* value |
| Gender |  |  |  |  |
| Male | Reference |  | Reference |  |
| Female | 1.43(1.23,1.65) | <0.001 | 0.82 (0.68, 0.98) | 0.031 |
| Age groups, year |  |  |  |  |
| ≤24 | Reference |  | Reference |  |
| 25 to 34 | 1.49 (1.37, 1.61) | <0.001 | 1.58 (1.45, 1.72) | <0.001 |
| 35 to 44 | 2.10 (1.91, 2.30) | <0.001 | 2.22 (1.99, 2.48) | <0.001 |
| >45 | 2.80 (2.53, 3.10) | <0.001 | 3.03 (2.67, 3.44) | <0.001 |
| BMI, kg/m^2^, n (%) |  |  |  |  |
| 18.5 to 24.9 | Reference |  | Reference |  |
| ≤18.4 | 1.87 (1.69, 2.08) | <0.001 | 2.18 (1.95, 2.44) | <0.001 |
| 25 to 29.9 | 0.75 (0.68, 0.83) | <0.001 | 0.66 (0.60, 0.73) | <0.001 |
| >30 | 0.65 (0.51, 0.81) | <0.001 | 0.68 (0.54, 0.86) | 0.002 |
| Missing | 1.01 (0.94, 1.09) | 0.704 | 0.90 (0.84, 0.98) | 0.011 |
| Marital status |  |  |  |  |
| Single | Reference |  | Reference |  |
| Married or cohabitating | 1.57(1.46,1.68) | <0.001 | 0.97 (0.89,1.06) | 0.540 |
| Divorced or separated | 1.71(1.50,1.93) | <0.001 | 1.14 (0.99,1.32) | 0.061 |
| Widowed | 1.94(1.29,2.90) | 0.002 | 0.98 (0.64,1.50) | 0.926 |
| Infection type |  |  |  |  |
| Homosexual | Reference |  | Reference |  |
| heterosexual | 1.57(1.44,1.72) | <0.001 | 1.39 (1.24,1.56) | <0.001 |
| PWID | 1.22(0.85,1.75) | 0.283 | 1.15 (0.78,1.69) | 0.495 |
| other | 3.40(2.34,4.93) | <0.001 | 2.00 (1.34, 2.98) | <0.001 |
| Year of diagnosis |  |  |  |  |
| Before 2014 | Reference |  | Reference |  |
| 2014 to 2016 | 0.65 (0.60, 0.70) | <0.001 | 0.56 (0.52, 0.61) | <0.001 |
| After 2016 | 0.64 (0.60, 0.69) | <0.001 | 0.47 (0.43, 0.51) | <0.001 |
| Lag time groups, month |  |  |  |  |
| 6 to 12 | Reference |  | Reference |  |
| ≤1 | 1.69(1.48,1.93) | <0.001 | 2.23 (1.93, 2.57) | <0.001 |
| 1 to 6 | 1.39(1.21,1.60) | <0.001 | 1.56 (1.35,1.81) | <0.001 |
| >12 | 1.24(1.06,1.45) | <0.001 | 1.09 (0.93, 1.28) | 0.301 |
| HCV/HBV seropositive |  |  |  |  |
| No | Reference |  | Reference |  |
| Yes | 1.37 (1.21, 1.54) | <0.001 | 1.24 (1.09, 1.41) | 0.001 |
| Tuberculosis |  |  |  |  |
| No | Reference |  | Reference |  |
| Yes | 14.26 (8.91, 22.83) | <0.001 | 11.73 (7.25, 18.96) | <0.001 |
