# Supplementary material for: Factors Associated With Delayed and Late Initiation of Antiretroviral Therapy Among Patients With HIV in Beijing, China, 2010–2020
Source: Int J Public Health. 2023 Jun 21;68:1605824. doi: 10.3389/ijph.2023.1605824 (PMC10321558; doi:10.3389/ijph.2023.1605824)
Supplement: Supplementary file 2 [file Table2.DOCX]

Table 2. Univariate and multiple regression analysis of delayed ART initiation. Beijing, 2010-2020

| Delayed ART initiation (>30 days) | | | | |
| --- | --- | --- | --- | --- |
|  | Univariate logistic regression | | Multiple logistic regression | |
| Covariate | OR (95%CI) | *P* value | OR (95%CI) | *P* value |
| Gender |  |  |  |  |
| Male | Reference |  | Reference |  |
| Female | 0.71 (0.61, 0.83) | <0.001 | 0.75 (0.62, 0.91) | 0.004 |
| Age groups, year |  |  |  |  |
| ≤24 | Reference |  | Reference |  |
| 25 to 34 | 0.96 (0.89, 1.03) | 0.218 | 0.95 (0.88, 1.03) | 0.252 |
| 35 to 44 | 0.81 (0.74, 0.88) | <0.001 | 0.87 (0.78, 0.97) | 0.014 |
| >45 | 0.60 (0.55, 0.66) | <0.001 | 0.82 (0.72, 0.94) | 0.004 |
| BMI, kg/m^2^ |  |  |  |  |
| 18.5 to 24.9 | Reference |  | Reference |  |
| ≤18.4 | 0.88 (0.79, 0.97) | 0.014 | 0.86 (0.76, 0.97) | 0.016 |
| 25 to 29.9 | 0.93 (0.85, 1.01) | 0.090 | 1.04 (0.95, 1.16) | 0.396 |
| >30 | 0.88 (0.72, 1.08) | 0.225 | 1.19 (0.96, 1.49) | 0.119 |
| Missing | 1.32 (1.23, 1.41) | <0.001 | 0.82 (0.76, 0.89) | <0.001 |
| Marital status |  |  |  |  |
| Single | Reference |  | Reference |  |
| Married or cohabitating | 0.75 (0.70, 0.80) | <0.001 | 0.77 (0.70, 0.84) | <0.001 |
| Divorced or separated | 0.69 (0.61, 0.78) | <0.001 | 0.88 (0.76, 1.03) | 0.113 |
| Widowed | 0.55 (0.36, 0.84) | 0.006 | 0.71 (0.43, 1.15) | 0.160 |
| Infection type |  |  |  |  |
| Homosexual | Reference |  | Reference |  |
| Heterosexual | 0.81 (0.74, 0.89) | <0.001 | 1.06 (0.93, 1.19) | 0.395 |
| PWID | 7.00 (4.25, 11.52) | <0.001 | 6.53 (3.84, 11.10) | <0.001 |
| Other | 1.32 (0.92, 1.91) | 0.132 | 0.81 (0.54, 1.21) | 0.307 |
| Year of diagnosis* |  |  |  |  |
| Before 2014 | Reference |  | Reference |  |
| 2014 to 2016 | 0.38 (0.35, 0.41) | <0.001 | 0.33 (0.30, 0.36) | <0.001 |
| After 2016 | 0.10 (0.09, 0.11) | <0.001 | 0.082 (0.075, 0.089) | <0.001 |
| CD4 count at ART initiation, /μL |  |  |  |  |
| >500 | Reference |  | Reference |  |
| ≤200 | 0.70 (0.64, 0.77) | <0.001 | 0.51 (0.46, 0.57) | <0.001 |
| 201 to 350 | 1.03 (0.94, 1.12) | 0.524 | 0.72 (0.65, 0.79) | <0.001 |
| 351 to 500, | 1.06 (0.96, 1.16) | 0.239 | 0.88 (0.80, 0.98) | 0.018 |
| Clinical manifestation of HIV |  |  |  |  |
| No | Reference |  |  |  |
| Yes | 0.92 (0.81, 1.05) | 0.240 | Not included |  |
| Opportunistic infection |  |  |  |  |
| No | Reference |  | Reference |  |
| Yes | 0.90 (0.79, 1.03) | 0.132 | 0.58 (0.50, 0.69) | <0.001 |
| HCV/HBV seropositive |  |  |  |  |
| No | Reference |  | Reference |  |
| Yes | 1.31 (1.16, 1.47) | <0.001 | 1.14 (0.99, 1.30) | 0.062 |
| Tuberculosis |  |  |  |  |
| No | Reference |  | Reference |  |
| Yes | 2.57 (1.83, 3.62) | <0.001 | 2.06 (1.40, 3.04) | <0.001 |
