# Supplementary material for: Factors Associated With Delayed and Late Initiation of Antiretroviral Therapy Among Patients With HIV in Beijing, China, 2010–2020
Source: Int J Public Health. 2023 Jun 21;68:1605824. doi: 10.3389/ijph.2023.1605824 (PMC10321558; doi:10.3389/ijph.2023.1605824)
Supplement: Supplementary file 3 [file Table3.DOCX]

Table 3. Univariate and multivariate regression analysis of either late or delayed ART initiation. Beijing, 2010-2020

| Either late or delayed ART initiation | | | | |
| --- | --- | --- | --- | --- |
|  | Univariate logistic regression | | Multiple logistic regression | |
| Covariate | OR (95%CI) | *P* value | OR (95%CI) | *P* value |
| Gender |  |  |  |  |
| Male | Reference |  | Reference |  |
| Female | 0.98 (0.84,1.14) | 0.761 | 0.76 (0.62, 0.93) | 0.006 |
| Age groups, year |  |  |  |  |
| <24 | Reference |  | Reference |  |
| 25-34 | 1.22 (1.14, 1.32) | <0.001 | 1.25 (1.16, 1.36) | <0.001 |
| 35-44 | 1.32 (1.21, 1.45) | <0.001 | 1.45 (1.30, 1.62) | <0.001 |
| >45 | 1.34 (1.21, 1.48) | <0.001 | 1.83 (1.60, 2.09) | <0.001 |
| BMI, kg/m^2^ |  |  |  |  |
| 18.5 to 24.9 | Reference |  | Reference |  |
| ≤18.4 | 1.31 (1.17, 1.47) | <0.001 | 1.43 (1.26, 1.61) | <0.001 |
| 25 to 29.9 | 0.81 (0.74, 0.88) | <0.001 | 0.83 (0.75, 0.91) | <0.001 |
| >30 | 0.70 (0.58, 0.86) | 0.001 | 0.90 (0.73, 1.12) | 0.349 |
| Missing | 1.31 (1.22, 1.41) | <0.001 | 0.85 (0.78, 0.92) | <0.001 |
| Marital status |  |  |  |  |
| Single | Reference |  | Reference |  |
| Married or cohabitating | 1.07 (1.00, 1.14) | 0.035 | 0.82 (0.74,0.90) | <0.001 |
| Divorced or separated | 1.10 (0.97, 1.26) | 0.142 | 1.05 (0.90,1.23) | 0.509 |
| Widowed | 0.79 (0.52, 1.19) | 0.252 | 0.59 (0.37,0.93) | 0.024 |
| Infection type |  |  |  |  |
| Homosexual | Reference |  | Reference |  |
| heterosexual | 1.11 (1.01, 1.22) | 0.035 | 1.24 (1.10,1.41) | 0.001 |
| PWID | 6.44(3.38, 12.28) | <0.001 | 4.73 (2.43,9.22) | <0.001 |
| other | 2.79 (1.71, 4.57) | <0.001 | 1.24 (0.72,2.13) | 0.430 |
| Year of diagnosis |  |  |  |  |
| Before 2014 | Reference |  | Reference |  |
| 2014 to 2016 | 0.29 (0.26, 0.32) | <0.001 | 0.29 (0.26, 0.32) | <0.001 |
| After 2016 | 0.10 (0.095, 0.12) | <0.001 | 0.10 (0.092, 0.11) | <0.001 |
| HCV/HBV seropositive |  |  |  |  |
| No | Reference |  | Reference |  |
| Yes | 1.51 (1.32, 1.72) | <0.001 | 1.25 (1.08, 1.45) | 0.002 |
| Tuberculosis |  |  |  |  |
| No | Reference |  | Reference |  |
| Yes | 82.38 (11.53, 588.64) | <0.001 | 42.80 (5.95, 308.14) | <0.001 |
